# Eosinophils and Bacteria, the Beginning of a Story

**DOI:** 10.3390/ijms22158004

**Published:** 2021-07-27

**Authors:** Edna Ondari, Esther Calvino-Sanles, Nicholas J. First, Monica C. Gestal

**Affiliations:** LSU Health, Department of Microbiology and Immunology, Louisiana State University (LSU), Shreveport, LA 71103, USA; edna.ondari@lsuhs.edu (E.O.); esthercalvino@outlook.com (E.C.-S.); nicholas.first@lsuhs.edu (N.J.F.)

**Keywords:** eosinophils, bacteria, Th1 responses, Th2 responses, microbiota, homeostasis

## Abstract

Eosinophils are granulocytes primarily associated with T_H_2 responses to parasites or immune hyper-reactive states, such as asthma, allergies, or eosinophilic esophagitis. However, it does not make sense from an evolutionary standpoint to maintain a cell type that is only specific for parasitic infections and that otherwise is somehow harmful to the host. In recent years, there has been a shift in the perception of these cells. Eosinophils have recently been recognized as regulators of immune homeostasis and suppressors of over-reactive pro-inflammatory responses by secreting specific molecules that dampen the immune response. Their role during parasitic infections has been well investigated, and their versatility during immune responses to helminths includes antigen presentation as well as modulation of T cell responses. Although it is known that eosinophils can present antigens during viral infections, there are still many mechanistic aspects of the involvement of eosinophils during viral infections that remain to be elucidated. However, are eosinophils able to respond to bacterial infections? Recent literature indicates that *Helicobacter pylori* triggers T_H_2 responses mediated by eosinophils; this promotes anti-inflammatory responses that might be involved in the long-term persistent infection caused by this pathogen. Apparently and on the contrary, in the respiratory tract, eosinophils promote T_H_17 pro-inflammatory responses during *Bordetella bronchiseptica* infection, and they are, in fact, critical for early clearance of bacteria from the respiratory tract. However, eosinophils are also intertwined with microbiota, and up to now, it is not clear if microbiota regulates eosinophils or vice versa, or how this connection influences immune responses. In this review, we highlight the current knowledge of eosinophils as regulators of pro and anti-inflammatory responses in the context of both infection and naïve conditions. We propose questions and future directions that might open novel research avenues in the future.

## 1. Eosinophils in Context

The body’s most powerful defense against external insults is the immune system. Orchestration of the intricate web of signals necessary to mount an adequate response to such insults requires finely-tuned mechanisms that mediate the function and activation of different immune cells [1]. Mucosal-associated organs and surfaces, such as the respiratory [2] or digestive tracts [3,4], have the greatest exposure to foreign antigens. This heightened susceptibility to infection requires intact, highly regulated, and precise mucosal responses. A key mediator of the maintenance of mucosal homeostasis and immunity are eosinophils [5].

Eosinophils are granulocytes with a segmented nucleus. Their dense granules contain cationic proteins, which include the major basic protein (MBP), eosinophil peroxidase (EPX), eosinophil cationic protein (ECP), and the eosinophil derived neurotoxin (EDN) [6,7,8]. The versatility of these granulocytes in homeostasis and immunity is gradually becoming better understood [9]. While they can present antigen [10], and regulate T cell responses [10] their most well-studied role to date is in eosinophil-mediated T_H_2 responses, including anti-helminthic activity [11,12,13]. This association with immunopathological states, such as allergy [14], asthma [15], and other chronic inflammatory disorders [16,17,18,19], is characterized partly by the positive feedback of T-cell expression of type-2 cytokines or the lack of anti-inflammatory responses that suppress inflammation when it is not needed. Despite being primarily associated with T_H_2 responses, eosinophils are also known to play a role in the clearance of viral respiratory infections during which they trigger T_H_1 immune responses [20,21]. This apparent dichotomy only reveals the lack of understanding of the actual role of these cells, as well as their biological functions during immune responses and pathological states. Although their functions in the gastrointestinal tract have been well described [22,23,24], there are limited studies on the role of eosinophils in bacterial infections, particularly at other mucosal sites.

This review aims to gather what is known about eosinophils and their cross-signals with bacteria in the context of infection and microbiota. We will briefly discuss what is known about their role during parasitic and viral infections to guide us on our hypothesis about their function during bacterial infections. Drawing insights from what is known about their role in gastrointestinal immunity, we aim to understand their respiratory system functions better. We discuss the mechanisms by which they modulate T cell responses and then conclude with perspectives and future directions that would highlight the gaps in the knowledge in this area of investigation.

## 2. Eosinophils and Innate Immunity

### 2.1. Eosinophils during Helminth Infections

Helminth infections are associated with elevated eosinophil counts and marked upregulation of T_H_2 cytokines, such as IL4, IL5, and IL-13 [11,25,26]. This T_H_2 milieu enables eosinophil infiltration to sites of infection and supports their survival and anti-parasitic activity [11]. In many human and murine helminth infection studies, such as schistosomiasis and trichinosis, eosinophils have been demonstrated to have direct parasiticidal effects [27,28], or promote worm expulsion [29], which further enhances T_H_2 responses. Synergistic effects of IL-4 on the anti-parasitic response include IgM to IgE class switching [30], reduced egg burden, and a subsequent decrease in morbidity [31]. In certain models of parasite infection, however, the absence of eosinophils or neutralization of IL-5 did not increase susceptibility to parasites, raising the mystery about the function of these cells [32,33]. Ablation of eosinophils elevated T_H_2 responses to nematode infection [32,33,34], and the presence of eosinophils blunted nitric-oxide-mediated parasite killing [35]. These observations suggest complex immunoregulatory mechanisms for eosinophils in the context of parasitic infections as well as parasite and niche-specific anti-parasitic mechanisms opening new questions about the evolutionary role of these relatively new cells during infections.

An undesired consequence of eosinophil-mediated immunity is dysregulated activity during chronic helminth infection, which results in pathology. Humans are often definitive hosts for large, multicellular parasites that have complex life cycles which undergo different stages/forms, often occupying multiple and sometimes immunologically inaccessible compartments within the body (such as the brain, liver, and muscle tissue), and thus present multiple antigens, and are often too large to expel or phagocytize, and typically cannot be cleared without intervention. While eosinophils are known to be effective against some parasites, such as migrant nematode larval stages [11,36], they are often ineffective against all large parasitic organisms [37]. This not only presents a challenge to mounting adequate host immunity, but the direct consequences of eosinophil activity, either degranulative or tolerogenic, often result in tissue damage.

Eosinophils are implicated in pathogenesis due to helminth infection, such as loasis, filariasis, toxocariasis, and schistosomiasis [11]. They achieve anti-parasitic activity against larval organisms through degranulation, releasing reactive oxygen species, cationic peptides, other granular proteins, or complement-mediated attack [11]. However, an indirect consequence of tissue-level infiltration and release of these eosinophil effectors is host cell cytotoxicity and tissue damage, as local anti-parasitic activity is often nonspecific [38]. In the event of failure to clear parasites, they also induce tolerogenic responses that may confine pathogens and limit damage to the host, but these responses may also be associated with pathogenesis. For instance, egg deposition by *Schistosoma* spp. in tissues and organs induces the formation of granulomas caused by infiltration of granulocytes, macrophages, and lymphocytes that are deposited around the eggs [31]. Extremely high eosinophil numbers resulting from helminth infection can lead to eosinophil-mediated organ damage, such as severe dermal pathology [39], tissue fibrosis [40], and organomegaly [40].

Eosinophils also contribute to T_H_2 granuloma formation during bacterial pathogenesis in the event of failure to resolve the primary infection. *Mycobacterium tuberculosis* infection in the murine lung, for instance, causes enhancement of a type-2 cytokine profile due to increased eosinophil accumulation in the lung, which contributes to augmented granuloma formation [41]. As several cell types are involved in T_H_2 granuloma development (such as macrophages, neutrophils, mast cells and lymphocytes), eosinophils have a significant contribution to the maintenance of a local T_H_2 milieu, which enhances their formation and maintenance. Eosinophils also heighten bacterial morbidity by dampening pro-inflammatory responses. Chronic, recurrent salmonellosis is frequent during helminth co-infection, in part due to the promotion of an anti-inflammatory T_H_2 response during concomitant parasitic infection mediated by eosinophils. In an experimental murine model, responses to schistosome egg antigens during *Schistosoma mansoni* and *Salmonella Typhimurium* infections not only impaired T_H_1/T_H_17 responses but also caused significant eosinophilic granuloma formation and failure of bacterial clearance [42]. 

### 2.2. Eosinophils during Viral Infections

In 1999, Adamko et al. observed that sensitization to ovalbumin in guinea pigs before infection with parainfluenza caused a decrease of the viral content in the lungs. Interestingly, this effect was reversed by the antibody to IL-5, providing the first evidence in vivo for a role for eosinophils in promoting antiviral host defense [43]. However, it was not until 2007 that Phipps et al. demonstrated that eosinophils express Toll-like receptors (TLRs) to recognize viral nucleic acids [44] that eosinophils started to gather attention. Later, in 2017, Samarasinghe et al. demonstrated that eosinophils present antigen to CD8^+^ T cells during Influenza A Virus infections [9], providing a novel functional aspect to this versatile cell type associated with T_H_1 responses.

A very interesting mechanism by which eosinophils inhibit antiviral responses of airway cells is by suppressing interferons (INF-β and INF-λ) during rhinovirus infections while having no significant effects on the infectability of the virus. The results obtained suggest that reduced interferon responses could promote viral replication, causing an increase in inflammation that can lead airway obstruction and an exacerbation of symptoms in patients with asthma [45]. 

Other known mechanisms through which eosinophils protect the host against virus include the RNase activity of cytoplasmic granules, production of oxidant agents, extracellular traps, and release of antiviral type I cytokines [46]. Moreover, it has been observed that an increase in histamine production occurs during viral infection in some studies [47,48], presenting another possible mechanism for virus clearance [49].

The relationship between the levels of eosinophils in the host and the severity of viral infection is still unclear. On the one hand, asthmatics, presenting pulmonary eosinophilia, are less likely to suffer severe influenza infection [9], attributing a protective role for these cells. On the other hand, the evidence that associates the severity of COVID-19 in patients with eosinophil-associated diseases [50] is not fully understood. Recent evidence suggests that in asthmatic patients, a pre-existent absolute eosinophil count equal or greater than 150 cells/μL is somehow protective of mortality and morbidity associated with COVID-19, and the authors suggest that understanding the mechanism by which eosinophils can function as protective cells against COVID-19 can provide novel insights into the disease [51]. During SARS-CoV-2 infection, eosinopenia is observed in a significant percentage of hospitalized patients [52], and the levels of eosinophils present a clear increase from acute to recovery phases [53] suggesting that these cells play a key role during the critical stages of disease. SARS-CoV-2 infection promotes strong granulocyte-macrophage colony-stimulating factor (GM-CSF) responses (known to activate eosinophils) [53], and its severity is associated with the emergence of CD62L^+^ eosinophils in response to IFNγ and the upregulated expression of PD-L1 on circulating eosinophils [54], suggesting that in this context lung resident eosinophils will be dampening pro-inflammatory responses. Consequently, although levels of eosinophils have not been proven to be the cause of severe COVID-19, there is a clear correlation between the decrease in eosinophils and the worsening of the prognosis. This is a unique condition compared with other types of pneumonia [55], and in fact, it has been proposed that eosinophils levels could be used for COVID-19 diagnosis [52].

### 2.3. Eosinophils during Bacterial Infections

Archer and Hirsch, in 1963, demonstrated the phagocytic abilities of eosinophils using cells from horse blood and rat peritoneal exudates [56], and later this property was observed in eosinophils isolated from other animals [57]. In 1968 and 1969, human eosinophils were proven to phagocytose bacteria, too [58], albeit this process occurs at a lower efficiency than in neutrophils. Cohen performed more detailed studies, which revealed that eosinophils and gram-positive bacteria are in contact in vivo, suggesting that eosinophils might have antigen-presenting capabilities and their attraction to antigen-antibody aggregates indicated a potential role in immunological processes [58]. 

Studies on the bactericidal activity of eosinophils have mainly been conducted using *Escherichia coli* [59] and *Staphylococcus aureus* as biological models [60,61]. However, there is no consensus on the efficacy at which eosinophils can neutralize bacteria when infected at high multiplicity of infection. Dechatelet et al. [62] show that while eosinophils and neutrophils have similar phagocytic rates. Indeed, eosinophils are less bactericidal due to the inability of eosinophil peroxidase to catalyze the peroxidase-H_2_O_2_-Cl-reactions and cannot clear bacteria in the absence of neutrophils [62]. In contrast, Yazdanbakhsh et al. [63] did not find significant differences between the bactericidal activity of neutrophils and eosinophils. Albeit, other reports showed that eosinophils are less capable of perforating the membrane of *E. coli* than neutrophils [63]. 

A complementary mechanism by which eosinophils exert direct pathogen immunity is through the formation of extracellular DNA traps, which consist of extruded DNA enmeshed with histones and cationic proteins [7,64,65,66]. Traps have been reported during gastrointestinal bacterial infections [22], where they have been associated with localized infection containment [67]. Charcot–Leyden crystal protein (CLC-P), also known as galectin-10, has been closely associated with eosinophils traps and may also be linked to their formation [68,69]. Extracellular traps have been correlated with several autoinflammatory and infectious diseases [7], although their role during immune response and pathogenesis is not fully clear. In addition to their phagocytic capabilities and extracellular trap formation, there is increasing evidence that associates eosinophils with immune responses to bacterial infections. 

Further proof corroborating the interaction of eosinophils with bacterial infection has been demonstrated in patients with eosinophilic conditions. In clinical studies, high peripheral eosinophil numbers have been associated with *Shigella* spp. infections, providing a potential biomarker for systemic disease [70]. Eosinophil counts are used as a prognostic marker in intensive care units [71,72,73,74] and are monitored during antibiotic treatment [75], as a prognostic marker for septicemia [76]. Patients with a type-2 allergy had increased protection against *S. aureus* septicemia. This mechanism is suggested to be mediated by eosinophils and innate lymphoid cells (ILC) 2, which promote an anti-inflammatory T_H_2 state [77]. Interestingly, treatment with the anti-IL-5-receptor antibody benralizumab, was shown to increase respiratory infections, especially by *Moraxella catarrhalis*, *Streptococcus pneumoniae*, *Staphylococcus epidermidis*, *Hemophilus influenza*, *Pseudomonas aeruginosa*, and metapneumovirus [78]. When examining the mechanism by which benralizumab exerts this effect on infections, while other anti-IL-5 treatments did not have similar effects, the authors discovered that benralizumab greatly reduced sputum eosinophilia [78], suggesting that eosinophils might play a role in innate responses [79]. Conversely, concomitant *Helicobacter pylori* has been shown to protect against eosinophilic esophagitis [80] and asthma [81], suggesting bacterial interaction with eosinophils to regulate the progression of eosinophilic disorders. This possess the question as to what the specific roles of eosinophils are under these conditions: are they participating in the immune responses to bacterial infections? Or do bacterial infections manipulate eosinophils to cause a hyper-reactive state that leads to eosinophilic disorders? In this context, a key area of investigation that has not been fully explored is respiratory diseases and eosinophilic disorders. Frequent respiratory infections in childhood are associated with an increase in the frequency of asthma [82,83] and allergies. A reported sequela of *Bordetella* spp. infections is the increased risk for asthma and allergies [84,85,86], which suggests that bacteria trigger this hyper-reactive eosinophilic state. However, recent literature has revealed that *B. bronchiseptica* can suppress eosinophil influx in the lungs to promote bacterial persistence [87], suggesting that eosinophils, in fact, actively participate in the immune responses to *Bordetella* spp. infections. These seemingly contradictory findings only increase the complexity of the puzzle that tries to answer the question, what is it that eosinophils do?

## 3. Eosinophils and T-Cell Immunity

Eosinophils are key modulators of adaptive immunity and maintenance of immune homeostasis. They interact with T_H_1, T_H_2, and T_H_17 T-lymphocyte subsets. Briefly, T_H_1 cells are involved in pro-inflammatory responses that mediate immunity to intracellular bacteria and viruses [88] and generally implicate many granulocytes. In contrast, T_H_2 responses involve the defense against large extracellular eukaryotic pathogens, such as helminths and some species of fungi, and are commonly associated with eosinophilic states [89,90]. Finally, T_H_17 responses are critical for the clearance of extracellular pathogenic bacteria and fungi [91] and are implicated in autoimmunity and inflammation [92,93]. 

Although eosinophils are primarily associated with the parasitic and anti-inflammatory responses as previously discussed, interestingly, eosinophils also mediate the generation of Mucosal Associated Lymphoid Tissue (MALT) tissue as a response to *H. pylori* infection via APRIL [94]. This finding demonstrates that the functions of eosinophils are more complex than we can imagine. In this section, we will discuss the functions of eosinophils in the inflammatory responses and the cumulative evidence of the eosinophil effector functions during gastric infections in the orchestration of the adaptive responses with the goal of seeding the idea that eosinophils play critical roles during bacterial infections that are yet to be discovered. 

### 3.1. Eosinophils and T_H_2 Immunity

**Figure 1:**
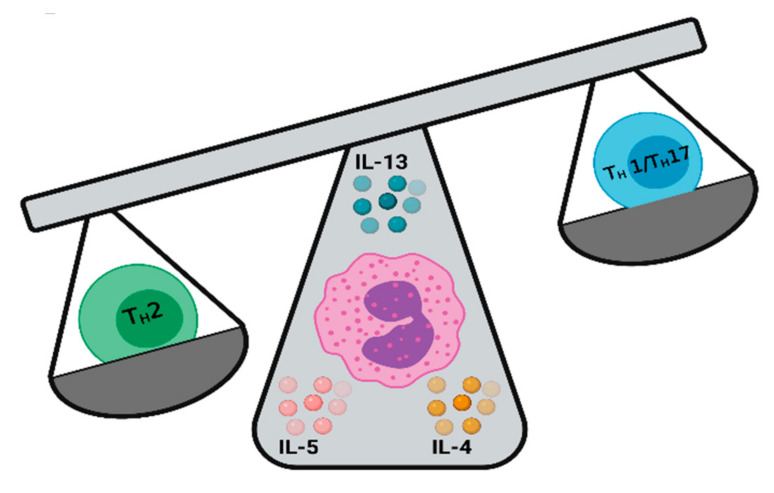
Eosinophils promote T_H_2 responses. Eosinophils responding and secreting cytokines, such as IL-4, IL-5, IL-13 and others, generates a type II immune response that can promote the generation of T_H_2 responses.

Eosinophils are essential modulators of T cell immunity. They establish T_H_2 responses by regulating T-helper lymphocyte differentiation and clonal proliferation, polarization, recruitment, activation, and function in response to specific stimuli, such as allergens and parasites [6]. In addition, they synthesize and secrete classical type 2 cytokines, including IL-4, IL-5, IL-6, IL-10, and IL-13 skewing the immune response towards a T_H_2 phenotype (Figure 1) [95,96]. Type 2 polarizing cytokines induce the expression of GATA-3 [97] and STAT-6 [98], essential transcription factors for T_H_2 lymphocyte differentiation and function. Eosinophils can also express major histocompatibility complex (MHC) class II and co-stimulatory molecules including CD80, CD86, CD9, CD28, CD40, and FcεRI [99,100,101,102], allowing them to process and present allergens [103] and antigens, such as those from helminths [10], as well as to direct specific T-cell proliferation, and trigger T_H_2 cytokine release [104].

T_H_2-associated eosinophil activity also mediates chronic respiratory immunopathology. In human atopic conditions and mouse challenge models of allergic airway inflammation, sensitization by allergens causes acute inflammatory responses and mast cell degranulation [105,106,107]. Subsequent activation of T_H_2 lymphocytes combined with eotaxin-1 and IL-5 release cause eosinophil activation, differentiation, and infiltration into the airway and facilitate their prolonged survival in this site, triggering recurrent airway hyperresponsiveness [108,109]. Degranulation of eosinophils in the airway induces damage to epithelia and surrounding tissue leading to chronic inflammation of the lung mucosa and airway remodeling in severe disease [110,111,112]. 

The critical role of eosinophils during these T_H_2 responses associated with parasites and eosinophilic immunopathology is undoubted, but eosinophils also contain other effectors and cytokines associated with pro-inflammatory responses. Therefore, the question is, can eosinophils stimulate pro-inflammatory responses during infections?

### 3.2. Eosinophils and T_H_1/T_H_17

**Figure 2:**
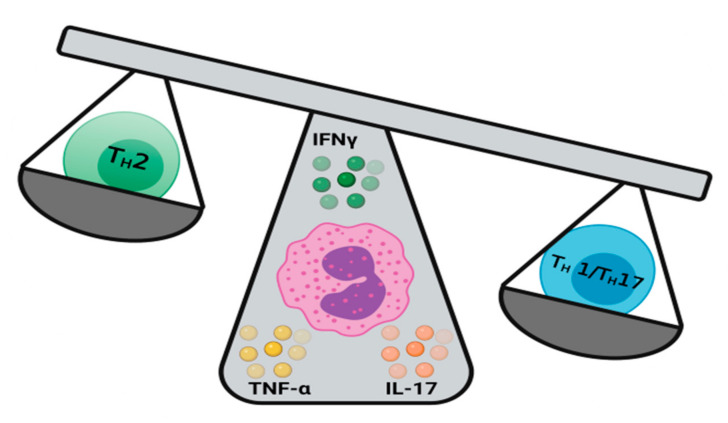
Eosinophils promote T_H_1/T_H_17 responses. Eosinophils contain and secrete cytokines, such as IFNγ, TNFα, IL-17, and others, generates a type I or type III immune response against external insults, such as viral infections.

While they are predominantly associated with T_H_2 responses, eosinophils also secrete T_H_1 cytokines, such as IFN-γ, TGF-β, IL-2, IL-8, IL-17, and IL-12 (Figure 2) [20,113,114,115]. Expression of T_H_1 cytokines by eosinophils is thought to be constitutive [20]. However, their secretion has been demonstrated to be differential or transient [20,115] and dependent on co-stimulation by CD28, CD86 [113], or other T_H_1 cytokines, such as TNF-α [115,116]. Thus far, eosinophil mediated T_H_1 responses have mainly been associated with the clearance of viral upper respiratory tract infections. Eosinophils can present viral antigens to T-cells via MHC class I, as previously demonstrated [117], triggering T-cell proliferation and IFN-γ release. Preformed cytokines, such as IL-2, IL-12, and IFN-γ within eosinophil granules are released in response to upper airway viral challenge [9,21], inducing a subsequent antiviral T_H_1 response.

Moreover, and further supporting a role of eosinophils to mount the proper adaptive response, eosinophils stimulate the development of tertiary lymphoid tissues via APRIL-secretion, and although eosinophils block pro-inflammatory responses during *H. pylori* infections, the involvement of these cells during the formation of tertiary lymphoid tissue suggests that they can enhance pro-inflammatory responses [23,94] associated with pro-tumorigenesis. During *Clostridium difficile* infections, IL-25 mediated eosinophilia has been shown to be protective, resulting in reduced severity and mortality by protecting the epithelial barrier during chronic inflammatory disorders in the mouse model.

Although eosinophils are known to suppress T_H_1 responses to counteract pro-inflammatory signals in the gastrointestinal tract during bacterial infection, which has been shown to be a critical step in the establishment and persistence of *H. pylori* infections, recent evidence suggests eosinophils promote T_H_17 responses in the lungs to clear bacterial infection [22]. In a murine model of respiratory infection, eosinophils promote T_H_17 responses in the lungs to clear *Bordetella bronchiseptica.* Mice lacking eosinophils develop a long-term persistent respiratory infection due to a failure during the generation of adaptive immune responses [22]. Based on these results, Gestal et al. have proposed a novel role for eosinophils in promoting pro-inflammatory responses during bacterial lung infections [87], suggesting a shift in the classical view of eosinophils as T_H_2 associated cells, even during bacterial infections. 

Taken together, this data suggests that eosinophils mediate immunity or pro-tumorigenic states of inflammation during *H. pylori* infections and that establishment of infection relies on the eosinophils’ ability to dampen the pro-inflammatory response. In the respiratory tract, eosinophils promote early resolution of infection, enhancing T_H_17 mucosal responses and leading to rapid clearance of the pathogen. Does this mean that bacteria have mechanisms that manipulate eosinophils to promote long-term chronic infections? This current evidence is just the beginning, exposing how critical, complex, and elusive this cell type is.

## 4. Eosinophils as Keepers of the T_H_1/T_H_2 Balance

**Figure 3:**
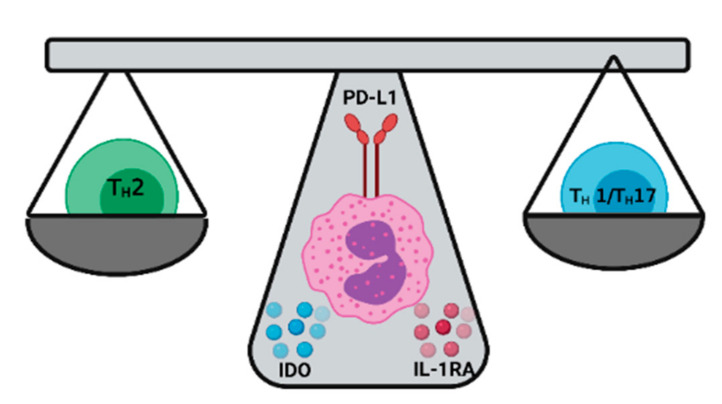
Eosinophilic maintenance of T_H_1/T_H_2 balance. Eosinophilic expression of IDO, PD-L1, and IL-1RA maintains the homeostasis between type I and type II immune responses. Generally, the secretion of these molecules is to control over-reactive pro-inflammatory responses.

The association of eosinophils and T_H_2 inflammatory responses is undoubted, but the specific mechanisms of action during parasitic infections and auto-inflammatory diseases are not fully defined yet. The LIAR hypothesis [118] provides a complete view of the eosinophils as regulators of the local immunity and remodeling/repair, encompassing a broader perspective of the eosinophilic functions. The LIAR hypothesis states that “eosinophils are part of host recognition pathways that identify focal bursts of cell death accompanied by cell proliferation, including possibly a mechanism for detecting local stem cell activities in outlying tissues/organs”. This provides a function for eosinophils in the tissues where remodeling is frequent, such as mammary glands, endometrium, organ transplant rejection, or cancers [118]. This would also make sense in the context of the infection process, during which many cells dye, and the influx of pro-inflammatory cells cause tissue fibrosis that will require remodeling. From this perspective, eosinophils oversee the upkeep of the balance between T_H_1 and T_H_2 responses to maintain the equilibrium and function of the organ and the mucosal tissue homeostasis (Figure 3).

In line with this hypothesis, steady-state numbers of these cells are present in organs, such as the lung, gastrointestinal tract, mammary gland, uterus, and thymus, which may imply these cells as principally tissue-resident in most mucosal tissues [119]. Moreover, eosinophils are associated with the maintenance of M2 alternatively activated macrophages to upkeep glucose homeostasis in adipose tissue [120] and production of GM-CSF by ILC3s has been shown to promote eosinophil activation and augmented cytokine secretion in colitis pathogenesis [121]. Eosinophils play the dual role of mediating pathogen or allergen-triggered T_H_1 or T_H_2 immunity and maintain T-cell homeostasis, often preventing potentially pathological imbalances of T-helper cell immune responses. While they can induce both pro-inflammatory and anti-inflammatory states, they predominantly skew the T-cell response by either promoting T_H_2 or actively dampening T_H_1 or T_H_17 responses under pro-inflammatory conditions.

As suppressors of over-reactive pro-inflammatory T_H_1-mediated spillover, eosinophils produce and secrete indoleamine 2, 3-dioxygenase (IDO) under the influence of IFN-γ [122,123,124]. IDO is produced by different immune cells, including eosinophils, monocytes, macrophages, and dendritic cells, and it catalyzes the oxidative metabolism of tryptophan to kynurenines that promote T_H_1 cell-specific apoptosis [122,125,126], leading to the long-term predominance of T_H_2 effector cell population at this site. It is known that eosinophils promote T_H_2 responses via IDO in asthma [122], allergy [127], cancer [128], and immune development [129,130]. This mechanism is critical, especially in early development, where the balance is more skewed towards T_H_2 [130]. IDO signaling is currently being investigated for cancer therapies, highlighting its crucial immunomodulatory role associated with the LIAR hypothesis. In fact, some types of cancer cells constitutively express IDO, providing exciting targets for novel immunomodulatory therapies [131,132].

Another mechanism by which eosinophils turn down pro-inflammatory responses is via programmed death-ligand 1 (PD-L1). In the gastrointestinal tract, IFNγ production during bacterial infection increases PD-L1 expression by eosinophils, suppressing T_H_1 proliferation [22]. Contact of PD-1 with PD-L1 can provoke T cell exhaustion, characterized by the loss of effector functions, decreased proliferation, and apoptosis [129]. PD-L1 is produced by many cells, including dendritic cells, monocytes, macrophages, and eosinophils, and has critical functions during bacterial infections. The net result of prolonged inflammation during sepsis is sustained PD-1/PD-L1, impairing innate and adaptive immune function [133] due to accelerated pro-inflammatory cell death. Anti-PD-L1 antibody treatment improves and maintains T cell numbers and function and significantly improves survival during burn-associated infection [134]. Similarly, during gastrointestinal infections with *Helicobacter pylori*, eosinophil suppresses T_H_1 responses by PD-L1 and INFγ mediated mechanisms. Indeed, *H pylori* lacking type 4 secretion systems, one of the major virulence factors, failed to induce T_H_1-cell suppression by eosinophils, as it does in the wild-type strain [22]. 

A third mechanism by which eosinophils control the over-reactive T_H_17 responses is via inhibition of the IL-1R1 signaling pathway [135]. In the intestine, eosinophils have been demonstrated to suppress IL-1β-mediated T_H_17-cellular differentiation and IL-17 production by secreting an IL-1 receptor antagonist (IL-1RA) [136]. The IL-1 family plays a vital part in T_H_17 differentiation, and IL-1RA has a critical role during this balance. IL-1RA is secreted mainly by eosinophils and epithelial cells [136]. In vivo studies using IL-1rn deficient mice found that excess IL-1 signaling enhances the development of T_H_17 cells, increasing the severity of asthma [137,138], providing a function for IL-1RA to prevent pathological stages of inflammation. Regulation of T_H_17 responses by eosinophils, however, is dichotomous and seems to be site or antigen-specific, as they have been shown to promote IL-1β/T_H_17 inflammation in the airway [139,140,141], and allergic inflammatory skin conditions [142,143], indicating that the classic idea that eosinophils promote T_H_2 responses might be an oversimplification of the many functions that this versatile cell may have. 

Taken together, these observations suggest that eosinophils have multiple roles in the balance between inflammatory and anti-inflammatory responses, which implies that eosinophils are not only mediators of anti-helminth immunity or hyper-reactive pathological states but are also keepers of the balance between pro and anti-inflammatory responses. The extent to which bacterial infections affect eosinophil function, however, is still not fully understood.

### 4.1. Eosinophils and Microbiota

#### 4.1.1. Eosinophils and Gut Microbiota

Eosinophils are most abundant in the gastrointestinal tract, which contains the most commensal bacterial biomass in the body [144,145]. Most eosinophils reside within the lamina propria in the gut and migrate to this site following an eotaxin gradient, amplified and sustained by IL-5 [22,146]. Previous mouse experiments established that migration of eosinophils to the gastrointestinal tract occurs mainly during fetal development, independently of intestinal microbiota [147]. Eosinophils interact closely with intestinal microflora and are essential for gut homeostasis by maintaining normal resident gastrointestinal microbial populations to enable normal epithelial barrier function. 

Interestingly, germ-free (GF) mice show a more significant proportion of eosinophils than specific pathogen-free controls, suggesting that microbiota can also suppress the uncontrolled proliferation of eosinophil populations [144]. Moreover, the complexity of the microbiota is also critical in the control of eosinophils numbers, and GF mice colonized by complex microbiota revealed a significant decrease in eosinophilia [144]. In contrast, colonization with simple microbiota does not cause a measurable decrease in eosinophils numbers. Lack of commensal bacteria also correlates with lower cytokine and chemokine production, except IL-3 and CXCL9, which both increase, suggesting an underdeveloped mucosal immune system; moreover, morphological changes in eosinophils are also noticeable in these conditions [148]. Consequently, despite the microbiota-independent migration of eosinophils to the GI tract, microbiota colonization affects turnover, activation state, and phenotype of eosinophils, indicating a feedback loop mechanism by which microbiota and eosinophils are interconnected. An example of phenotypic change due to microbiota colonization is an increase in eosinophil subpopulations characterized by higher expression levels of SiglecF and CD11c, similar to that produced in inflammatory responses [149]. However, the molecular mechanism responsible for this interaction and cross signaling is still not fully understood. 

**Figure 4:**
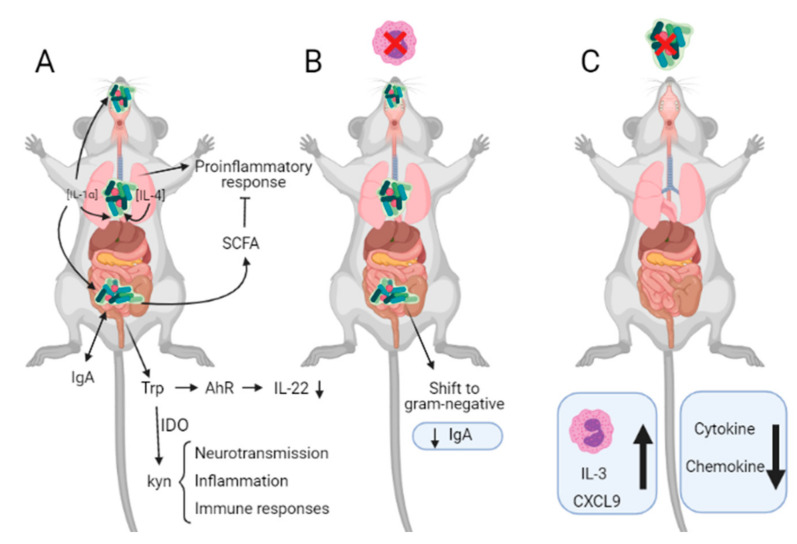
Cross-signaling between eosinophils and microbiota. (**A**) Summary of some processes in which murine microbiota is involved. (**B**) Alterations observed in eosinophil-deficient mice. (**C**) Alterations observed in germ-free (GF) mice.

Metabolism of tryptophan is controlled directly or indirectly by microbiota, and it can follow two metabolic pathways that affect immune response in different mechanisms [130]. On the one hand, it can be transformed into kynurenine by a process that is mediated by IDO. The activity of IDO is stimulated by microbiota, and it is involved in neurotransmission, inflammation, and immune responses. On the other hand, gut microbiota can promote the conversion of tryptophan into ligands of the aryl hydrocarbon receptor (AhR), which is an important component of the immune response at the barrier site and correlates with increased production of IL-22 (Figure 4) [150].

The fluidity of both microbiota and eosinophils modulates each other in the gut. Microbiota comprises potential pathogens whose overgrowth is known to be regulated by eosinophils. Eosinophil-deficient mice showed an altered microbiota composition with a shift to gram-negative bacteria and a low production of IgA [151]. A possible mechanism that can explain this shift in microbial communities in the gut might be associated with the expression of MyD88, which is one of the primary mechanisms to control the overgrowth of gram-positive bacteria and stimulates IgA production [152]. Moreover, it is worth noting that eosinophils restrict T_H_1 responses against microbiota and are an essential source of inducible nitric oxide synthase (iNOS), which catalyzes the synthesis of antibacterial reactive nitrogen intermediates [153].

Nevertheless, how do eosinophils control microflora growth? It is well known that this cell type is required for the maintenance of intestinal IgA^+^ plasma cells and supports IgA class switching, an antibody that regulates gut microbiota. A feedback loop has been suggested between IL-1β expressing eosinophils and IgA to be involved in gut homeostasis [153,154]. It can be inferred that the mutual interaction between microbiota and eosinophils exists. Eosinophils interact closely with intestinal microflora and are essential for gut homeostasis by maintaining normal resident gastrointestinal microbial populations to enable normal epithelial barrier function. Moreover, microbiota and eosinophils could work synergistically during the immune response [148]. 

One example of this well-synchronized interaction happens during *Clostridium difficile* infection. During infection, levels of IL-25 significantly decrease, which is associated with an enhancement of type 2 responses, while increasing levels of IL-23, which promotes T_H_17 responses via IL-17. Mice treated with exogenous IL-25 were protected from lethal infection, an effect that was dependent on the influx of CD11b+SiglecF+ eosinophils to the gut [155]. However, more work will need to be done to better understand how these signals allow the microbiota-immune system, and especially eosinophils, to communicate. Although most investigations focus on microbiota isolated from stool samples, significant differences exist between the microbiota inhabiting different niches within the gut [156]. Taken together with the fact that microorganisms can live even under the most extreme environmental conditions on the earth, this evidence provides insight into the importance of exploring the different niches available in the human body, not only the gut, to provide a more communal understanding of the impact of microbial communities in our physiology [157].

#### 4.1.2. Eosinophils and Lung Microbiota

Recent studies have reported the existence of microbiota in the lungs of healthy mice, a niche that, until recently, was thought to be sterile [158]. Moreover, a negative correlation between the concentration of IL-1α and the diversity of microbiota in the lung has been found [159]. This is supported by the fact that in the lungs of wild-type mice, resident eosinophils (rEOS) are not present at birth, increasing gradually until day 7 [160]. Previous reports have associated this delay in establishing a resident population of eosinophils in the lungs with the development of microbiota [160]. To further support this relationship, house dust mite (HDM)-induced eosinophilia, a disease that is commonly associated with neonate mice, the microbiota population show a significant proportion of Firmicutes and Gammaproteobacteria, both related to asthmatic phenotype in humans and mice [161]. 

In addition to the interaction between microbiota and eosinophils within the lung, studying different human body niches is vital due to the evidence of bidirectional interaction between lungs and the gut. For example, short-chain fatty acids (SCFA) derived from gut bacteria inhibit pro-inflammatory responses in the lungs [157], demonstrating that the effects of gut microbiota are not specific for that anatomical site. Additionally, a connection between the concentration of IL-4 in the lungs and the diversity of oral and cecal bacterial communities has been reported [159]. It is also essential to keep in mind that microbiota is composed of bacteria and includes fungi, viruses, and archaea. Antifungal treatment can affect bacterial and fungal communities, leading to increased type 2 allergic airway inflammation and eosinophilia in an HDM model [157]. The human body and its microbiota have coevolved for millions of years. Consequently, perturbations in this community can affect immune response [152], so the effect of microbiota in infectious diseases requires further investigation. 

## 5. Conclusions

The eosinophilic paradigm depicts these cells as hyper-reactive effectors whose functions trigger harmful asthmatic and allergic reactions. Likewise, there is common knowledge of their immune activities during parasitic infections, particularly those caused by helminths, and their role in local immunity and remodeling/repair. However, a shift in perspective recognizes these cells for their traditional roles and presents a new idea posing eosinophils as immunomodulators that respond to bacteria. Interestingly there is evidence of eosinophils promoting anti-inflammatory responses during *Helicobacter pylori* infections in the guts and protective barrier responses during *Clostridium difficile* chronic inflammatory bowel disorder; while on the contrary, eosinophils promote pro-inflammatory responses in the lung following infection with a *Bordetella bronchiseptica* mutant. Technological advances and growing interests have revealed these critical roles of these cells, not only during T_H_2 responses, but also as keepers of the homeostatic balance between pro and anti-inflammatory responses that need to be cautiously orchestrated during bacterial infections, especially those that are highly persistent, such as *H. pylori* or *Bordetella* spp., and that can end in a fatal prognosis.

Nevertheless, eosinophils are not alone, and we should consider our body as a universe where microbes, fungi, viruses, phages, and cells, share niches, communicate, orchestrate, and overall dictate the physiological status of each individual. The new possibilities opening with the significant advances in the OMICS fields are only starting to demonstrate how all the signals are interconnected, resulting in a pathological state. However, many questions remain unanswered, is microbiota regulating eosinophils or vice versa? How do microbiota and eosinophils communicate? What is the role of eosinophils during health and disease? This last is a question that has already been proposed by many highly relevant eosinophils experts [8,118,162,163,164,165], and excitingly, there is still a lot that needs to be known by this fascinating and enigmatic cell offering exciting opportunities to investigate.

## Abbreviations

MBP	Major basic protein
EPX	Eosinophil peroxidase
ECP	Eosinophil cationic protein
EDN	Eosinophil derived neurotoxin
TLR	Toll-like receptor
CLC-P	Charcot-Leyden crystal protein
ILCs	Innate lymphoid cells
IDO	Indoleamine 2, 3-Dioxygenase
IFNγ	Interferon γ
PD-L1	Programmed death-ligand 1
GM-CSF	Granulocyte-macrophage colony-stimulating factor
IL-1Ra	IL-1 receptor antagonist
GF	Germ free
SCFA	Short-chain fatty acids
Kyn	Kynurenine
AhR	Aryl hydrocarbon receptor
